# Knee complaints vary with age and gender in the adult population. Population-based reference data for the Knee injury and Osteoarthritis Outcome Score (KOOS)

**DOI:** 10.1186/1471-2474-7-38

**Published:** 2006-05-02

**Authors:** Przemyslaw T Paradowski, Stefan Bergman, Anne Sundén-Lundius, L Stefan Lohmander, Ewa M Roos

**Affiliations:** 1 Department of Orthopedics, Clinical Sciences, Lund University Hospital, SE-221 85 Lund, Sweden; 2 The Department of Reconstructive Surgery and Arthroscopy of the Knee, Medical University Hospital, Zeromskiego 113, PL-90-549 Lodz, Poland; 3 Spenshult Hospital for the Rheumatic Diseases, SE- 313 92 Oskarström, Sweden; 4 Department of Physical Therapy, Health Sciences, Lund University Hospital, SE-221 85 Lund, Sweden

## Abstract

**Background:**

Self-reported knee complaints may vary with age and gender. Reference data from the adult population would help to better interpret the outcome of interventions due to knee complaints. The objectives of the present study were to describe the variation of self-reported knee pain, function and quality of life with age and gender in the adult population and to establish population-based reference data for the Knee injury and Osteoarthritis Outcome Score (KOOS).

**Methods:**

Population-based cohort retrieved from the national population register. The knee-specific Knee injury and Osteoarthritis Outcome Score (KOOS) was mailed to 840 subjects aged 18–84 yrs.

**Results:**

68% response rate. Women in the age group 55–74 reported more knee-related complaints in all the KOOS subscales than age-matched men. The differences were significant for the subscales *Pain *(p = 0.027), *Symptoms *(p = 0.003) and *ADL *function (p = 0.046).

In men, worse *ADL *and *Sport and Recreation *function was seen in the oldest age group 75–84 years compared to the younger age groups (p < 0.030). In women, worse *Pain *(p < 0.007), *ADL *(p < 0.030), *Sport and Recreation *(p < 0.001) and *QOL *(p < 0.002) were seen already in the age group 55–74 compared to the younger age groups.

**Conclusion:**

We found pain and other symptoms, physical function, and knee-related quality of life to vary with age and gender implying the use of age- and gender matched reference values for improved understanding of the outcome after interventions due to knee injury and knee OA.

## Background

Disability of the knee is a common problem across the population. In most population-based epidemiological studies singe-item questions are used to estimate the prevalence of knee pain. To assess the outcome of interventions due to knee injury and knee osteoarthritis however, the use of multi-item knee-specific outcome measures giving a broader picture of the clinical status is recommended [1,2]. One such instrument is the Knee injury and Osteoarthritis Outcome Score (KOOS) which has been validated for anterior cruciate ligament reconstruction [3], meniscectomy [4] and total knee replacement [5], procedures performed in different age groups of the adult population. Several studies using other knee-specific outcome scores have shown that the average score for a control group rarely is equivalent to the best possible score and also indicated differences due to age and gender [6-8]. Thus, it is essential to establish reference data from the general population to determine the influence of demographic factors such as age and gender on the perceived self-reported knee status of patients, and consequently better determine the true impact of treatment strategies. There is a paucity of studies that investigate knee pain, knee function and knee-related quality of life across the adult population. Population based studies have this far focused on adults older than 50 years [7-9].

The objective of the present study was to investigate the variation of self-reported knee pain, function and quality of life with age and gender in the adult population and to establish population-based reference data for the Knee injury and Osteoarthritis Outcome Score (KOOS).

## Methods

### Design and data collection

A population-based sample was randomly chosen from the National Population Records for the region Skåne of Southern Sweden. All persons in Sweden are registered in the National Population Records which is updated every six weeks. Skåne holds approximately 1/9 of the Swedish population and include both urban and rural communities. A simple sampling method was used and the Knee Injury and Osteoarthritis Outcome Score (KOOS) was sent to 840 persons aged 18–84 years. Each of 7 age groups (18–24, 25–34, 35–44, 45–54, 55–64, 65–74, 75–84) consisted of 60 men and 60 women. The number chosen (60 men+60 women/10 year stratum) was based on experience from clinical studies using the KOOS where a clinically significant difference often is set to 10 points and standard deviations in the magnitude of 10-15-20 have been seen in different populations and at different time points following interventions. To find a clinically significant difference of 10 points (SD 20, p = 0.05, 80% power) between men and women within an age stratum, totally 120 subjects would be needed (60 M+60W).

No other characteristics besides age and gender were obtained. The KOOS questionnaire was mailed together with a covering letter explaining the purpose of the study, and a prepaid return envelope. The non-responders were reminded twice with the same covering letter as the first time and a new KOOS questionnaire and a new prepaid return envelope. The Research Ethics Committee at the Faculty of Medicine, Lund University, Sweden approved the study (LU 600–00).

### Questionnaire

Knee-specific complaints were obtained by the Swedish version LK 1.0 of the Knee Injury and Osteoarthritis Outcome Score (KOOS) [4]. The KOOS is a 42-item self-administered knee-specific questionnaire assessing pain (9 items), symptoms (7 items), activities of daily living (17 items), sport and recreation function (5 items) and knee-related quality of life (4 items) in five separate subscales (for the KOOS questionnaire see Additional file: 1). Each item is responded to by marking one of five response options on a Likert scale. The WOMAC LK 3.0 [10] items are included in the first three KOOS subscales. KOOS has been validated for short- and long-term follow-up studies of knee injury and OA [3-5]. KOOS was considered reliable and responsive for assessment of knee complaints in a recent comparative review of knee-specific outcome measures [11].

### KOOS scoring

All items were scored from 0 to 4 and summed. Raw scores were then transformed to a 0 to 100 scale where 100 represent the best result. A separate score was calculated for each of the five subscales. In accordance with the users guide [12], if the number of missing items was less than or equalled 2 in a subscale they were substituted by the average item value for that subscale. If more than two items of the subscale were omitted the response was considered invalid and no subscale score was calculated.

### Statistical analysis

The statistical analysis was performed with SPSS for Windows 12.0 software package (SPSS Inc., Chicago, IL, USA). To increase power and minimize the number of comparisons made, the originally 7 age groups were collapsed into 4 age groups (18–34, 35–54, 55–74, and 75–84) when testing for differences due to gender and age. Parametric methods were used. Some of the data is not normally distributed, but the sample size in each group is large enough to apply the central limit theorem which gives normally distributed sample means. Analysis of variance and Students' t-test with Bonferroni correction was used because of multiple comparisons.

## Results

**Figure 1:**
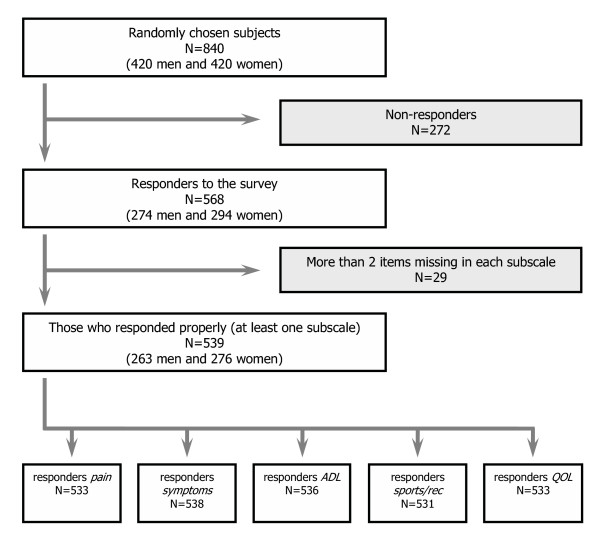
Flow chart that details the study procedure and formation of the patient cohort.

568 subjects (68%) responded to the questionnaire. For 29 persons, more than 2 items were missing for all subscales and no scores could be calculated. Scores for at least one subscale could thus be calculated for 539 subjects or 64% (65% for women and 63% for men), Figure 1. The number of subjects who responded varied with age. 54% responded in the youngest age group and 60% in the oldest one. The highest response rate (73%) was observed among those aged 65–74. Only in that age group did men respond more frequently than women (82% vs. 65%).

### Gender-related differences

**Figure 2:**
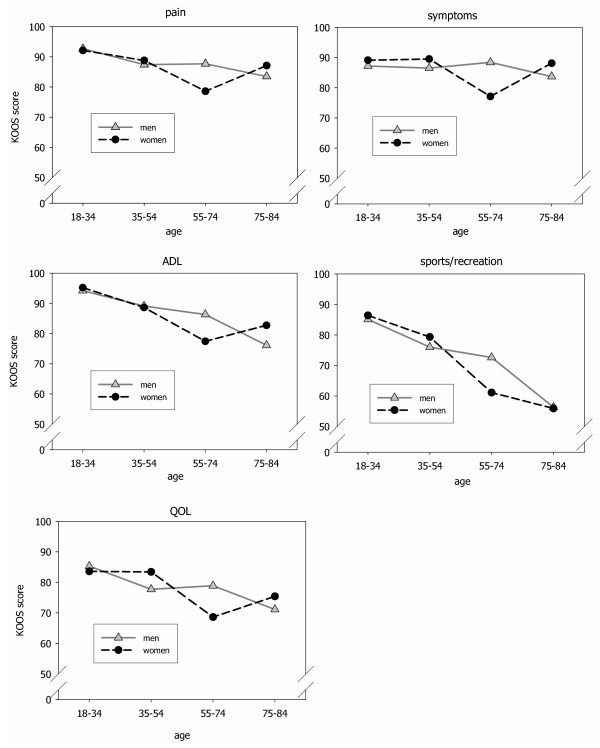
Mean KOOS scores of the subscales pain, symptoms, activities of daily living, sports/recreation, and quality of life for men and women in different age groups.

Women in the age group 55–74 reported more knee-related complaints in all the KOOS subscales than age-matched men, Table 1 and Figure 2. The differences were significant for the subscales *Pain *(p = 0.027), *Symptoms *(p = 0.003) and *ADL *function (p = 0.046).

### Age-related differences

Age-related differences were studied separately in men and women. In men, more difficulty was seen in the oldest age group 75–84 years compared to all the younger age groups for *ADL *function (p < 0.025), *Sport and Recreation *function (p < 0.030). For *QOL *more difficulty was seen in the oldest age group 75–84 years compared to the youngest age group 18–34, p = 0.045, Table 1 and Figure 2.

In women, the age group 55–74 years reported worse outcome compared to both the younger age groups in *Pain *(p < 0.007), *ADL *(p < 0.030), *Sport and Recreation *(p < 0.001) and *QOL *(p < 0.002). Women in the oldest age group 75–84 years reported worse outcome compared to the youngest women aged 18–34 in *ADL *function, and compared to both the younger age groups in Sport and Recreation function (p < 0.001).

## Discussion

To our knowledge this work is the first to evaluate the distribution and severity of knee complaints across the whole adult population as measured with a knee-specific outcomes measure. In the population, severity of clinically relevant knee complaints varies with age and gender.

Knee pain is common. In a study assessing general musculoskeletal pain, the 12-month prevalence of knee pain in the Dutch population 25 years old and over has been reported to be 21.9% [13]. Less is known of the functional limitations that may result from knee pain. In a British population sample aged 50 and over Jinks et al. found the 12-month period prevalence of all knee pain to be 47% [7]. In the same sample they also, by the use of a knee-specific questionnaire, found that 14% reported severe knee pain, 20% reported severe difficulty with at least one area of functioning and 12% reported both, indicating the importance of evaluating both function and pain.

We found functional difficulties to increase with age, supporting previous studies in the population and in knee patients [7-9,14,15]. A strength of our study is that we obtained data from adult subjects of a wide age range and thus could see that the previously noted deterioration in knee function in elderly is gradual during the whole adult life. Previous studies on knee pain and knee function in the population and in knee patients have focused on those over 50 years of age and have thus not been able to study these aspects [7-9,14,15].

The decline in function with older age groups was more apparent for the subscale Sport and recreation function compared to the subscale ADL function (Fig. 2). The subscale Sport and Recreation Function holds items representing more difficult lower extremity functions not required for activities of daily living as defined by the items of the KOOS subscale ADL. The Sport and Recreation subscale is thus more sensitive to reduction of lower extremity function, something frequently seen in clinical studies [16-19]. It has however been shown that these items are relevant for every other person undergoing Total Knee Replacement (mean age 71), indicating the relevance of this subscale also for older age groups [5].

The variation seen with age and gender may be due to both knee-specific and generic factors. A limitation of our study is that no data was collected on knee disease or general health status making it difficult to further explore the reasons for the variation seen. The prevalence of radiographic signs of OA increases with age which may partly explain our findings [20,21]. It is however unlikely that our results are explained by knee pathology only. In the population, musculoskeletal pain is more common in women than men [13]. Sex hormones, as well as psychosocial factors, are related to increased perception of pain in women compared to men [22]. The dramatically worse knee-related outcomes seen in our study in women in the age group 55–64 compared to women in the age group 45–54 may thus be related to menopause which occurs at a mean of 51.5 years [23].

Knee pain may also be part of a more widespread pain syndrome. The prevalence of widespread pain is clearly related to age with a significant increase in subjects over 50 years of age [24]. In a population study, long-standing knee pain in women was more often part of a widespread pain syndrome than knee pain in men (68% vs. 40%) [25]. In future studies, and in the clinic, it may be of value to assess the subject's total body pain in order to separate subjects with knee pain only from those where knee pain is part of a widespread pain syndrome.

The generally better knee status seen in women in the oldest age group (75–84) may support the role of both knee pathology and widespread pain as explanatory factors for the variation seen with age in women. The dominating knee pathology at this age is osteoarthritis. Knee replacement is the most effective treatment in reducing pain due to osteoarthritis, and about 90% report satisfactory pain relief [26]. According to the Swedish Knee Register's Annual Report from 2004 [27], the prevalence of total knee replacement is highest at 80 years and it can be estimated that every twentieth Swedish woman at age 80 has a knee replacement. In a population study, the prevalence of chronic widespread pain in women was highest at age 60–64 and then dropped with increasing age [25], indicating that factors not related to the knee may also contribute to the generally better knee status seen in women in the oldest age group.

We had a response rate in this study of 68% which is comparable to others [7,28]. A low response rate can also bias the overall results of pain prevalence estimates since people with chronic pain are more likely to respond [25]. Also, it has been shown that subjects with a previous history of knee problems have a tendency to respond to medical surveys more readily than those without [29]. The variation in response rate with age and gender could be a consequence of these two issues. The supposed higher incidence of chronic pain and previous knee problems amongst responders could lead to an overall overestimation of reported problems, but only to a minor extent affect the comparisons that the conclusions are based on. When performing the a priori power calculation we did not calculate with non-responders. Correctly, we should have calculated with 35–50% non-responders and thus included 35–50% more subjects into the study to, with sufficient power, detect differences between genders within each 10 year age stratum. To deal with this shortcoming, we collapsed the original 7 age strata into 4 wider age strata for analysis of differences due to age and gender. The reference data in this study is based on the response of 539 adult men and women. Increased precision of the confidence intervals of the means would require more subjects. The KOOS can to some extent be compared to the generic outcome measure SF-36, both instruments are scored on a 0–100 worst to best scale and the SF-36 holds subscales like Physical Function and Bodily Pain corresponding well to the KOOS subscales *ADL *and *Pain*. The Swedish normative data for the SF-36 is based on 8.930 persons [30]. For comparison, the 95% confidence interval for the mean of the SF-36 subscale Physical Function of women aged 20–24 (n = 889) is 1.6 points and for women aged 75–79 (n = 150) 10 points. For the comparable KOOS subscale ADL the 95% confidence interval of the mean for women aged 18–24 (n = 36) was 4.7 points and for women aged 75–84 (n = 34) 13.7 points. It can thus be estimated that at least a 10-fold larger population-based study sample than in the current study is required to decrease the confidence intervals for the KOOS subscale significantly. It should be a matter of discussion if this precision would improve interpretation of results in clinical studies.

## Conclusion

We found pain, physical function and knee-related quality of life to vary with age and gender implying the use of age- and gender matched reference values for improved understanding of the outcome after interventions due to knee injury and knee OA.

## Competing interests

The author(s) declare that they have no competing interests.

## Authors' contributions

PTP analyzed the data and drafted the manuscript. SB participated in the analysis of the data, contributed to the design of the study and revision of the manuscript, ASL collected the data, LSL contributed to the design of the study and revision of the manuscript, EMR contributed to the design of the study, participated in the analysis of the data, helped to draft the manuscript and revised the manuscript. All authors read and approved the final manuscript.

## Pre-publication history

The pre-publication history for this paper can be accessed here:



## Supplementary Material

Additional File 1 KOOS questionnaire Click here for file

## Figures and Tables

**Table 1:** Age-specific KOOS scores given as mean, standard deviation, median, (95%CI of the mean) for men (M) and women (W).

KOOS subscales	Mean score, SD, median (95%CI of the mean) in different groups							
	
	18–34		35–54		55–74		75–84	
	
	M	W	M	W	M	W	M	W
Pain	N = 60	N = 74	N = 78	N = 80	N = 88	N = 85	N = 34	N = 33
	92.2	92.1	87.4	88.8	87.7	78.6	83.5	87.1
	11.2	14.0	17.9	18.7	17.4	25.5	23.3	18.2
	97.2	97.2	97.2	97.2	97.2	91.7	94.4	96.9
	(89.8–95.6)	(88.8–95.3)	(83.4–91.5)	(84.6–93.0)	(84.0–91.4)	(73.1–84.1)	(75.3–91.6)	(80.6–93.5)
								
Symptoms	N = 60	N = 74	N = 78	N = 82	N = 88	N = 85	N = 36	N = 34
	87.2	89.1	86.5	89.5	88.4	77.1	83.7	88.1
	13.9	13.5	16.7	14.6	17.3	24.8	19.0	14.2
	92.9	92.9	92.9	95.8	96.4	85.7	87.5	94.6
	(83.6–90.8)	(86.0–92.2)	(82.7–90.2)	(86.2–92.7)	(84.8–92.1)	(71.7–82.4)	(77.3–90.1)	(83.1–93.0)
								
ADL	N = 60	N = 74	N = 78	N = 80	N = 88	N = 85	N = 36	N = 34
	94.2	95.2	89.1	88.6	86.3	77.4	76.1	82.7
	10.0	11.6	17.6	19.7	18.8	26.2	24.8	19.5
	100	100	100	98.5	97.1	91.2	83.1	91.9
	(91.6–96.7)	(92.5–97.8)	(85.1–93.1)	(84.2–92.9)	(82.3–90.3)	(78.8–83.1)	(67.7–84.5)	(75.9–89.6)
								
Sport/Rec	N = 60	N = 74	N = 76	N = 80	N = 87	N = 84	N = 35	N = 34
	85.1	86.4	76.0	79.3	72.6	61.0	56.3	55.9
	20.8	21.1	29.5	27.7	29.9	36.9	34.7	37.3
	92.5	95.0	87.5	90.0	80	70.0	55.0	62.5
	(79.7–90.5)	(81.5–91.3)	(69.2–82.7)	(73.1–85.4)	(66.2–78.9)	(53.0–69.0)	(44.4–68.3)	(42.9–68.9)
								
QOL	N = 59	N = 74	N = 78	N = 80	N = 88	N = 85	N = 35	N = 33
	85.3	83.6	77.7	83.4	78.9	68.6	71.1	75.4
	19.2	20.2	25.4	22.0	25.4	31.4	29.0	26.9
	93.8	87.5	87.5	93.8	87.5	75.0	75.0	83.3
	(80.3–90.3)	(78.9–88.3)	(72.0–83.5)	(78.5–88.3)	(73.5–84.3)	(61.8–75.4)	(61.1–81.0)	(65.9–85.0)

## References

[B1] Altman R, Brandt K, Hochberg M, Moskowitz R, Bellamy N, Bloch DA, Buckwalter J, Dougados M, Ehrlich G, Lequesne M, Lohmander S, Murphy WA, Rosario-Jansen T, Schwartz B, Trippel S (1996). Design and conduct of clinical trials in patients with osteoarthritis: recommendations from a task force of the Osteoarthritis Research Society. Results from a workshop. Osteoarthritis Cartilage.

[B2] Bellamy N, Kirwan J, Boers M, Brooks P, Strand V, Tugwell P, Altman R, Brandt K, Dougados M, Lequesne M (1997). Recommendations for a core set of outcome measures for future phase III clinical trials in knee, hip, and hand osteoarthritis. Consensus development at OMERACT III. J Rheumatol.

[B3] Roos EM, Roos HP, Lohmander LS, Ekdahl C, Beynnon BD (1998). Knee Injury and Osteoarthritis Outcome Score (KOOS)–development of a self-administered outcome measure. J Orthop Sports Phys Ther.

[B4] Roos EM, Roos HP, Ekdahl C, Lohmander LS (1998). Knee injury and Osteoarthritis Outcome Score (KOOS)–validation of a Swedish version. Scand J Med Sci Sports.

[B5] Roos EM, Toksvig-Larsen S (2003). Knee injury and Osteoarthritis Outcome Score (KOOS) – validation and comparison to the WOMAC in total knee replacement. Health Qual Life Outcomes.

[B6] Demirdjian AM, Petrie SG, Guanche CA, Thomas KA (1998). The outcomes of two knee scoring questionnaires in a normal population. Am J Sports Med.

[B7] Jinks C, Jordan K, Croft P (2002). Measuring the population impact of knee pain and disability with the Western Ontario and McMaster Universities Osteoarthritis Index (WOMAC). Pain.

[B8] Bremner-Smith AT, Ewings P, Weale AE (2004). Knee scores in a 'normal' elderly population. Knee.

[B9] Jinks C, Jordan K, Ong BN, Croft P (2004). A brief screening tool for knee pain in primary care (KNEST). 2. Results from a survey in the general population aged 50 and over. Rheumatology (Oxford).

[B10] Bellamy N, Buchanan W, Goldsmith CH (1988). Validation study of WOMAC: A health status instrument for measuring clinically important patient-relevant outcomes following total hip or knee arthroplasty in osteoarthritis. Orthop Rheumatol.

[B11] Garratt AM, Brealey S, Gillespie WJ (2004). Patient-assessed health instruments for the knee: a structured review. Rheumatology (Oxford).

[B12] http://www.koos.nu.

[B13] Picavet HS, Schouten JS (2003). Musculoskeletal pain in the Netherlands: prevalences, consequences and risk groups, the DMC(3)-study. Pain.

[B14] Brinker MR, Lund PJ, Barrack RL (1997). Demographic biases of scoring instruments for the results of total knee arthroplasty. J Bone Joint Surg Am.

[B15] Ritter MA, Thong AE, Davis KE, Berend ME, Meding JB, Faris PM (2004). Long-term deterioration of joint evaluation scores. J Bone Joint Surg Br.

[B16] Roos EM, Roos HP, Ryd L, Lohmander LS (2000). Substantial disability 3 months after arthroscopic partial meniscectomy: A prospective study of patient-relevant outcomes. Arthroscopy.

[B17] W-Dahl A, Toksvig-Larsen S, Roos EM (2005). A 2-year prospective study of patient-relevant outcomes in patients operated on for knee osteoarthritis with tibial osteotomy. BMC Musculoskelet Disord.

[B18] von Porat A, Roos EM, Roos H (2004). High prevalence of osteoarthritis 14 years after an anterior cruciate ligament tear in male soccer players – A study of radiographic and patient-relevant outcomes. Ann Rheum Dis.

[B19] Roos EM, Roos HP, Lohmander LS (1999). WOMAC Osteoarthritis Index–additional dimensions for use in subjects with post-traumatic osteoarthritis of the knee. Western Ontario and MacMaster Universities. Osteoarthritis Cartilage.

[B20] Verbrugge LM (1995). Women, men, and osteoarthritis. Arthritis Care Res.

[B21] Felson DT, Nevitt MC (1999). Estrogen and osteoarthritis: how do we explain conflicting study results?. Prev Med.

[B22] Fillingim RB (2000). Sex, gender, and pain: women and men really are different. Curr Rev Pain.

[B23] McKinlay SM, Bifano NL, McKinlay JB (1985). Smoking and age at menopause in women. Ann Intern Med.

[B24] Thomas E, Peat G, Harris L, Wilkie R, Croft PR (2004). The prevalence of pain and pain interference in a general population of older adults: cross-sectional findings from the North Staffordshire Osteoarthritis Project (NorStOP). Pain.

[B25] Bergman S, Herrstrom P, Hogstrom K, Petersson IF, Svensson B, Jacobsson LT (2001). Chronic musculoskeletal pain, prevalence rates, and sociodemographic associations in a Swedish population study. J Rheumatol.

[B26] Robertsson O, Dunbar M, Pehrsson T, Knutson K, Lidgren L (2000). Patient satisfaction after knee arthroplasty: a report on 27,372 knees operated on between 1981 and 1995 in Sweden. Acta Orthop Scand.

[B27] http://www.ort.lu.se/knee/.

[B28] Croft P, Lewis M, Wynn Jones C, Coggon D, Cooper C (2002). Health status in patients awaiting hip replacement for osteoarthritis. Rheumatology (Oxford).

[B29] Baker P, Reading I, Cooper C, Coggon D (2003). Knee disorders in the general population and their relation to occupation. Occup Environ Med.

[B30] Sullivan M, Karlsson J (1994). SF-36 Hälsoenkät: Swedish Manual and Interpretation Guide.

